# The correlation between pain and intrinsic capacity in older patients with chronic diseases: a cross-sectional study

**DOI:** 10.3389/fpubh.2026.1822852

**Published:** 2026-07-15

**Authors:** Wei Cui, Yihong Lu, Lini Dong, Jia Guo, Fengjiao Li

**Affiliations:** 1 Clinical Nursing Teaching and Research Section, The Second Xiangya Hospital, Central South University, Changsha, Hunan, China; 2 Department of Geriatrics, The Second Xiangya Hospital, Central South University, Changsha, Hunan, China; 3 Cardiovascular Surgery Intensive Care Unit, The Second Xiangya Hospital, Central South University, Changsha, Hunan, China

**Keywords:** chronic diseases, healthy aging, intrinsic capacity, older adults, pain

## Abstract

**Objective:**

To explore the association between chronic pain and intrinsic capacity (IC) decline status in older patients with chronic diseases.

**Methods:**

A cross-sectional study was conducted among older patients with chronic diseases in a tertiary hospital from January 2021 to December 2024. Chronic pain was assessed through self-report and defined as pain persisting for ≥3 months. IC was evaluated across locomotor, vitality, cognitive, psychological, and sensory domains with validated tools. Logistic regression was used to examine the association between chronic pain and IC decline status.

**Results:**

A total of 820 participants were analyzed. Chronic pain prevalence was 75.3, and 87.0% were classified as having IC decline status. Locomotor and vitality were the most affected domains. Chronic pain was strongly associated with IC decline status (adjusted OR = 4.77, 95% CI: 3.04–7.49). Age ≥80 years increased the risk of IC decline status, while polypharmacy (≥5 medications) showed a protective effect. No significant interactions were found between chronic pain and age or polypharmacy.

**Conclusions:**

Chronic pain was highly prevalent and independently associated with IC decline status in older patients with chronic diseases. Locomotor and vitality were the most vulnerable domains. Integrating pain assessment and management with interventions targeting mobility and vitality may help preserve functional health in multimorbid older adults.

## 1 Introduction

The global population is aging at an unprecedented rate. The number of people aged 60 years and older is projected to reach 2.1 billion by 2050 (1, 2). In China, the number of individuals aged 60 and above has reached 264 million (3). Advances in medicine and public health have substantially extended life expectancy. However, current evidence remains insufficient to indicate that this extension has been matched by a parallel increase in healthy life expectancy (4). As life expectancy increases, a growing proportion of older adults spend additional years living with chronic conditions, such as cardiovascular diseases (5), diabetes (6), and chronic respiratory disorders (7). These conditions are often progressive and long-lasting, leading to cumulative functional decline and reduced quality of life (8).

In 2015, the World Health Organization (WHO) first introduced the concept of “Intrinsic Capacity” (IC) in its “World Report on Aging and Health”, defining it as “the composite of all physical and mental capabilities that an individual possesses” (9). IC refers to the total physical and mental reserves that determine an individual's ability to function. It includes five domains: cognition, psychological wellbeing, sensory function, vitality, and locomotion (10). Decline in IC may present as reduced mobility, poor balance, malnutrition risk, fatigue, cognitive impairment, depressive symptoms, and vision or hearing problems. Therefore, IC decline reflects broader functional vulnerability than disease diagnosis alone. It provides a basis for early identification of decline and for interventions that support healthy and active aging (11). A Chinese community-based study reported that 39.9% of older adults had intrinsic capacity decline (12). Understanding the factors that may influence IC has therefore become a key step in improving outcomes for older adults.

Chronic pain is very common among older adults. Previous research has shown that 37.8% of older adults report chronic pain (13). Despite its high prevalence, chronic pain often receives limited attention in clinical settings (14). It is a subjective experience, varies across individuals, and frequently coexists with multiple chronic conditions, which makes assessment and management challenging.

Persistent pain can limit physical activity, diminish muscle strength, and contribute to a decline in mobility (15). Chronic pain is also associated with muscle weakness and fatigue, gradually lowering overall vitality and energy reserves (16). Beyond these physical consequences, long-term pain is linked to emotional distress, depression, and anxiety, exerting a strong impact on psychological wellbeing (17, 18). Research has also suggested that chronic pain may interfere with attention, processing speed, and memory, contributing to gradual cognitive decline (19). Prolonged pain has been associated with changes in sensory processing, including altered stimulus perception and increased sensitivity, which may affect interaction with the surrounding environment (20). Chronic pain may affect multiple domains of health in older adults, which makes its relationship with IC an important area of investigation.

Studies have reported associations between chronic pain and impairments in mobility, mood, cognition, and daily activity performance (15–20). However, most research has examined these outcomes separately rather than within the integrated framework of IC. Limited evidence is available among older adults with chronic diseases, particularly in hospital-based settings in China. Therefore, it remains unclear whether chronic pain is associated with IC decline status and which IC domains are most affected in this population. Examining chronic pain within the WHO IC framework may provide a more comprehensive understanding of its relationship with multidimensional functional decline in older patients with chronic diseases.

This study aims to examine the relationship between chronic pain and IC in older adults with chronic diseases. It will assess how pain is associated with different domains of IC and whether it is associated with overall functional ability. The findings are expected to provide evidence for early intervention and guide care strategies that focus on maintaining function.

## 2 Materials and methods

### 2.1 Study design and participants

Participants were recruited between January 2021 and December 2024 at a tertiary grade-A hospital in Hunan Province, China. It was a cross-sectional observational study targeting older patients with chronic diseases. Convenience sampling was used to recruit eligible participants. Specifically, potential participants were identified in the geriatric ward by the head nurse in collaboration with the research team, based on pre-defined inclusion and exclusion criteria. Patients who met the eligibility criteria were approached during hospitalization and invited to participate in the study. The inclusion criteria were: (1) age ≥ 60 years; (2) diagnosis of at least one chronic disease by a physician; (3) being able to independently complete the survey or only requiring minimal assistance. Minimal assistance referred to limited support such as reading the questions aloud or recording responses, while all responses were provided by the participants themselves rather than by proxies. Patients with terminal-stage diseases and an expected survival period of ≤ 6 months were excluded.

The sample size was estimated using the following formula for cross-sectional studies: $n\;=\;\frac{Z_{\alpha }^{2}\cdot \;\left(1-p\right)}{d^{2}}$. Assuming a 95% confidence level (Z_α_ = 1.96), an estimated prevalence of 39.9% (12), a margin of error of 0.05, the theoretical sample size was 370. To compensate for an anticipated 10% of invalid questionnaires, the minimum required sample size was set at 412. A total of 836 older patients with chronic diseases were finally included in the study, meeting the required threshold.

### 2.2 Data collection

Data were collected using two approaches. Locomotor function and cognitive status were evaluated by trained healthcare professionals through standardized assessments, while demographic information, health behaviors, clinical status, pain, and other IC domains were obtained using self-administered questionnaires with unified instructions. All questionnaires were collected immediately after completion and reviewed on site for completeness. Any missing or inconsistent responses were verified and corrected directly with the participants. All data were entered into a secure database and independently double-checked by two researchers to ensure accuracy. To maintain data quality, all research staff received standardized training and strictly adhered to the unified assessment protocol.

### 2.3 Measurement

#### 2.3.1 General information questionnaire

Participants completed a structured questionnaire collecting general sociodemographic and behavioral information, including age group, sex, marital status, education level, living arrangement, smoking, drinking, fall history within the past 12 months, and polypharmacy (defined as taking five or more medications concurrently during hospitalization). All items were self-reported using standardized response options.

#### 2.3.2 IC

Based on the definition of IC by the WHO, each domain (locomotion, vitality, cognition, psychological, and sensory) was assessed using a domain-specific instrument with established validity, which is a common approach in the current intrinsic capacity literature (10). In this study, IC was assessed across five domains using standardized, validated simplified Chinese versions of the scales suitable for the domestic population. The locomotion domain was evaluated with the Short Physical Performance Battery (SPPB) (21), which assesses lower-extremity function through three components: standing balance, gait speed, and chair-stand performance. Total scores range from 0 to 12, with higher scores indicating better physical performance. Vitality was assessed using the Short-Form Mini Nutritional Assessment (MNA-SF) (22), which consists of six items covering food intake, weight loss, mobility, psychological stress or acute disease, neuropsychological problems, and body mass index. Scores range from 0 to 14, with higher scores indicating better nutritional status. Cognition was measured with the Mini-Mental State Examination (MMSE) (23), which evaluates orientation, registration, attention and calculation, recall, and language. A representative item asks participants to identify the current date or recall previously presented words. Psychological status was evaluated using the Patient Health Questionnaire-9 (PHQ-9) (24), a nine-item instrument measuring depressive symptoms over the previous 2 weeks. Representative items include “Little interest or pleasure in doing things” and “Feeling down, depressed, or hopeless.” Each item is scored from 0 to 3, yielding a total score of 0–27. In the present study, the Cronbach's α of PHQ-9 was 0.891. The sensory domain was assessed based on self-reported vision and hearing impairments that affected daily life. All instruments used in this study were validated assessment tools that have been widely applied in older adult populations.

Each impaired IC domain was assigned 1 point, yielding a total score ranging from 0 to 6. Participants with a total score of 0 were classified as having intact IC, whereas those with a score of ≥1 were classified as having IC decline status, indicating the presence of impairment in at least one IC domain (25). The assessment criteria and scoring of intrinsic capacity components are presented in Table 1.

**Table 1:** Assessment criteria and scoring of intrinsic capacity components.

Domain	Assessment criteria	Score
Locomotion	SPPB>9 points	Yes (0 points)/no (1 point)
Vitality	MNA-SF>11 points	Yes (0 points)/no (1 point)
Cognition	MMSE>24 points (for secondary school and above), >20 points (primary school), >17 points (illiterate)	Yes (0 points)/no (1 point)
Psychological	PHQ-9 < 5 points	Yes (0 points)/no (1 point)
Sensory	Self-reported vision impairment affecting daily life	Yes (0 points)/no (1 point)
	Self-reported hearing impairment affecting daily life	Yes (0 points)/no (1 point)

#### 2.3.3 Chronic pain

Chronic pain was self-reported and defined as pain persisting for ≥3 months, regardless of cause or location. Participants were asked, “Have you experienced chronic or persistent pain for at least 3 months?” (yes/no). This definition is consistent with the widely accepted criteria for chronic pain proposed by the International Association for the Study of Pain (IASP) and has been commonly applied in epidemiological studies (26).

### 2.4 Statistical analysis

Data were entered using Microsoft Excel and analyzed with SPSS version 27.0 (27). Continuous variables were expressed as mean ± standard deviation (x ± s), and categorical variables were presented as frequencies and percentages. Differences between groups were compared using the χ^2^ test. Before performing multivariate logistic regression analysis, multicollinearity among independent variables was assessed. Tolerance and variance inflation factor (VIF) from linear regression models were used as diagnostic criteria (28). Logistic regression models were established with stepwise adjustment for potential confounders to examine the association between chronic pain and IC. Interaction terms were included to explore whether chronic pain and other variables jointly influenced the decline of IC. *p* < 0.05 was considered statistically significant for all tests.

### 2.5 Ethics statement

This study was approved by the Institutional Review Board of the Second Xiangya Hospital of Central South University (No. 2022-074). All participants provided written informed consent before participation. The study was conducted in accordance with the Declaration of Helsinki.

## 3 Results

### 3.1 General characteristics of the participants

**Figure 1:**
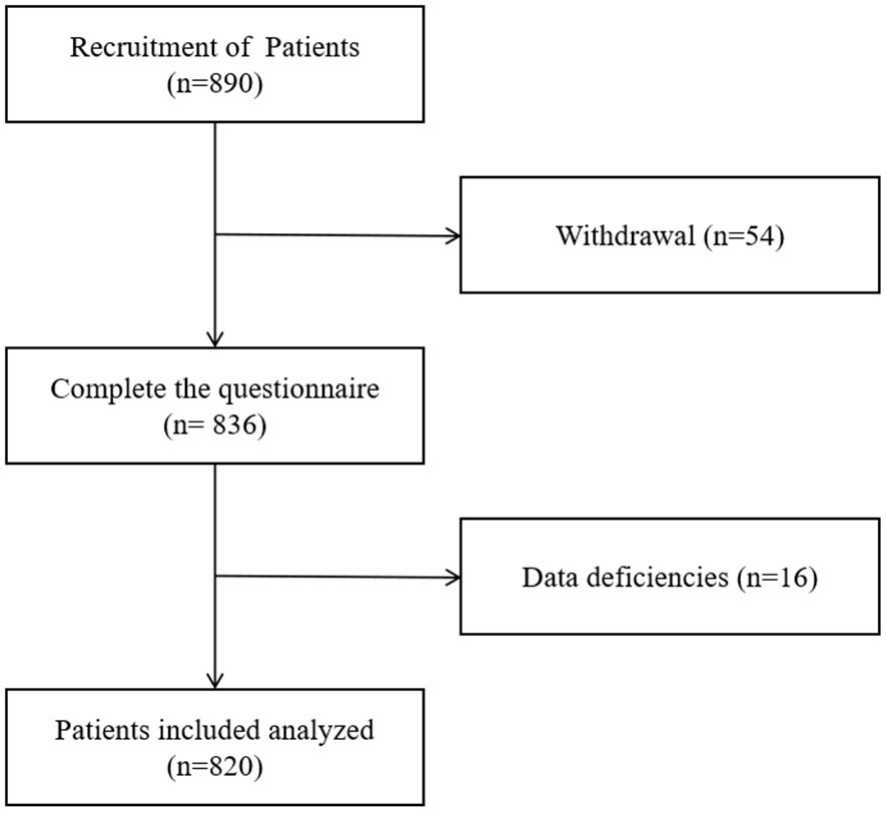
Flow diagram of participant recruitment and inclusion.

A total of 890 patients with chronic diseases were recruited, among whom 836 completed the questionnaire survey. There were 16 questionnaires with missing data. Ultimately, 820 questionnaires were included in the statistical analysis. The recruitment flowchart is shown in Figure 1.

As shown in Table 2, compared with participants with intact IC, those with IC decline status had statistically significant differences in age, smoking, drinking, polypharmacy, and recent history of falls. Additionally, there were significant differences in chronic pain. There were no significant differences between the two groups in terms of gender, marital status, education level, and living alone.

**Table 2:** Characteristics of participants by IC status (*n*, %).

Variables	Normal IC (*n* = 107)	Declined IC (*n* = 713)	χ^2^	*p*-values
Age group			18.406	< 0.001
60~79	96 (89.7)	498 (69.8)		
≥80	11 (10.3)	215 (30.2)		
Gender			2.627	0.105
Male	55 (51.4)	307 (43.1)		
Female	52 (48.6)	406 (56.9)		
Education level			1.522^1^	0.825
Illiteracy	3 (2.8)	34 (4.8)		
Primary school	23 (21.5)	170 (23.8)		
Middle school	23 (21.5)	138 (19.4)		
High school	31 (29.0)	182 (25.5)		
University and above	27 (25.2)	189 (26.5)		
Marital status			1.325	0.250
Widowed/unmarried	35 (32.7)	195 (27.3)		
Married	72 (67.3)	518 (72.7)		
Solitude			2.559	0.110
Yes	8 (7.5)	92 (12.9)		
No	99 (92.5)	621 (87.1)		
Smoking			7.215	0.007
Yes	17 (15.9)	201 (28.2)		
No	90 (84.1)	512 (71.8)		
Drinking			8.783	0.003
Yes	19 (17.8)	227 (31.8)		
No	88 (82.2)	486 (68.2)		
Polypharmacy			9.474	0.002
Yes	33 (30.8)	333 (46.7)		
No	74 (59.2)	380 (53.3)		
Falling history			4.793	0.029
Yes	19 (17.8)	198 (27.8)		
No	88 (82.2)	515 (72.2)		
Chronic pain			69.474	< 0.001
Yes	46 (43.0)	572 (80.2)		
No	61 (57.0)	141 (19.8)		

^1^Fisher test.

### 3.2 Chronic pain and IC level

As shown in Table 2, among the older patients with chronic diseases included in this study, the prevalence of chronic pain was as high as 75.3%. The overall proportion of IC decline status was 87.0%, suggesting a generally compromised functional status in this population. Further analysis of each domain showed that the highest decline rate was observed in locomotor function (66.6%), followed by vitality (40.5%), psychological health (18.2%), and cognitive ability (14.3%). Declines in vision and hearing were relatively less common, at 13.2 and 11.6%, as detailed in Table 3.

**Table 3:** Decline in each dimension of IC (*n*, %).

IC status	Mobility	Vitality	Cognition	Psychological health	Vision function	Hearing function	IC
Normal IC	274 (33.4)	488 (59.5)	703 (85.7)	671 (81.8)	712 (86.8)	725 (88.4)	107 (13.0)
Declined IC	546 (66.6)	332 (40.5)	117 (14.3)	149 (18.2)	108 (13.2)	95 (11.6)	713 (87.0)

### 3.3 Collinearity diagnosis

The results of collinearity diagnostics showed that all independent variables considered for inclusion in the logistic regression model had VIF values less than two and tolerance values greater than 0.7, indicating no significant multicollinearity. Detailed statistics are provided in Table 4.

**Table 4:** Collinearity diagnostics for independent variables.

Variables	Tolerance	VIF
Age group	0.982	1.018
Smoking	0.792	1.263
Drinking	0.802	1.246
Polypharmacy	0.976	1.025
Falling history	0.953	1.049
Chronic pain	0.928	1.077

### 3.4 Regression analysis of the association between chronic pain and IC decline status

To investigate the association between chronic pain and IC decline status in older adults with chronic diseases, a series of four logistic regression models (Model 0–Model 3) were constructed by stepwise adjustment for different categories of potential confounders. The outcome variable was IC decline status (yes/no), and the results are summarized in Table 5. Model 0 included only chronic pain. Model 1 adjusted for demographic factors (age). Model 2 further included health behavior variables (smoking, drinking). Model 3 added disease-related variables (polypharmacy, fall history).

**Table 5:** Logistic regression analysis of chronic pain and IC decline status.

Variables	Model 0		Model 1		Model 2		Model 3	
	OR (95%CI)	*p*-values	OR (95%CI)	*p*-values	OR (95%CI)	*p*-values	OR (95%CI)	*p*-values
Chronic pain	5.380 (3.518, 8.227)	<0.001	5.430 (3.525, 8.363)	<0.001	4.970 (3.202, 7.714)	<0.001	4.772 (3.040, 7.490)	<0.001
Age			3.833 (1.981, 7.417)	<0.001	3.930 (2.026, 7.622)	<0.001	3.670 (1.885, 7.143)	<0.001
Smoking					0.795 (0.413, 1.396)	0.375	0.789 (0.425, 1.462)	0.451
Drinking					0.613 (0.344, 1.093)	0.097	0.605 (0.337, 1.087)	0.093
Polypharmacy							0.595 (0.347, 0.948)	0.029
Falling history							0.819 (0.467, 1.435)	0.485

Reference categories: chronic pain, no; age, 60–79 years; smoking, no; drinking, no; polypharmacy ≤ 5 medications; fall history = no.

Chronic pain remained a significant independent risk factor in all models. Age ≥80 years was also consistently associated with the risk of IC decline status. Polypharmacy (≥5 medications) showed a protective effect in the fully adjusted model (Model 3). Smoking, drinking, and fall history were not statistically significant.

### 3.5 The interaction between chronic pain and other variables on the decline of IC

As shown in Table 6, after introducing the interaction terms “chronic pain × age group” and “chronic pain × polypharmacy” into the model, the results of the Logistic regression analysis showed that neither of the two interaction effects reached statistical significance.

**Table 6:** Logistic regression analysis of interaction effects.

Variables	*B*	SE	wald	OR (95%CI)	*p*-values
Chronic pain	1.848	0.284	42.213	6.346 (3.634, 11.082)	<0.001
Age	1.321	0.472	7.834	3.748 (1.486, 9.452)	0.005
Polypharmacy	−0.792	0.344	5.292	0.453 (0.231, 0.889)	0.021
Chronic pain _x_Age	−0.101	0.676	0.022	0.904 (0.240, 3.398)	0.881
Chronic pain _x_Polypharmacy	−0.466	0.470	0.985	0.627 (0.250, 1.575)	0.321

## 4 Discussion

This study revealed a high prevalence of chronic pain and a substantial decline in IC among older patients with chronic diseases. Chronic pain was reported by 75.3% of participants, which is markedly higher than the 57.4% prevalence reported in previous studies of older adults with chronic conditions (29). This discrepancy may reflect differences in study populations, as our sample included patients from a tertiary hospital setting where disease burden and symptom severity are typically greater. The proportion of participants with IC decline status reached 87.0%, which is consistent with previous studies (30). Logistic regression analysis further showed that chronic pain was independently associated with IC decline status, with an adjusted odds ratio of 4.77, suggesting that pain may be closely related to functional vulnerability within the IC framework.

Beyond the overall prevalence of IC decline status, domain-specific analysis provides further insight into the functional profile of this population. Locomotor impairment emerged as the most affected area, which is consistent with prior studies identifying mobility decline as one of the earliest markers of IC decline status and overall frailty in older adults (31). Decline in vitality ranked second only to locomotor impairment in this study, a pattern that is consistent with previous research (12). However, the proportion of participants with reduced vitality in our study was markedly higher than that reported in community-based studies (12). This discrepancy may be explained by the characteristics of the hospital-based sample, where patients often present with greater disease burden, higher rates of multimorbidity, and increased nutritional risk compared to community-dwelling older adults. Locomotion and vitality were the two most affected domains, suggesting that declines in physical performance and physiological reserve may represent the primary manifestations of reduced intrinsic capacity in this population.

Among participants with IC decline status in this study, the proportion of impairment in the psychological domain was higher than that observed in the cognitive domain. This may be because older adults with chronic diseases are more likely to experience depressive symptoms and emotional distress related to long-term disease burden and functional limitations, whereas cognitive impairment often develops more gradually (32). These findings underscore the importance of screening for psychological wellbeing in older adults with chronic diseases, as mental health changes may emerge earlier and interact with physical decline to accelerate overall loss of IC. The prevalence of self-reported declines in vision and hearing in this study was lower than that reported in other studies using objective sensory assessments, which may reflect differences in measurement methods and the potential underestimation of subclinical impairments through self-report (33).

Chronic pain was strongly associated with overall IC decline status. Rather than affecting a single function, chronic pain may contribute to multidimensional impairments involving locomotion, vitality, and psychological capacity, thereby accelerating the loss of functional reserves. Previous research demonstrated that pain affects multiple physiological and psychological systems simultaneously, resulting in cumulative effects on overall capacity (34, 35). Several mechanisms may underlie this relationship. Chronic pain can restrict physical activities and daily mobility, resulting in the deterioration of physical function and a reduction in physiological recovery capacity (36). Furthermore, it may provoke persistent stress responses and psychological distress, potentially impairing the ability to cope with health-related challenges (37). The effect of chronic pain on IC decline status did not vary by age or medication status, indicating that its influence on overall functional reserve was relatively consistent across these subgroups. Consequently, integrating comprehensive pain assessment and management into the care of chronic conditions may be essential to safeguard functional capacity and support healthy aging.

Advanced age was associated with a higher risk of IC decline status, supporting the concept that cumulative physiological changes and reduced adaptability with aging progressively weaken functional reserves in older adults (38). In contrast, polypharmacy showed a protective association in the fully adjusted model, a finding that differs from much of the existing literature (39, 40). However, the observed protective association of polypharmacy should be interpreted with caution. This finding may reflect differences in medication management, healthcare access, or residual confounding in this hospital-based population, rather than a direct protective effect of polypharmacy itself.

This study has several limitations. First, this was a cross-sectional study, and therefore causal relationships between chronic pain and intrinsic capacity cannot be established. In addition, the term “IC decline” in this study refers to a cross-sectional status of impaired intrinsic capacity, rather than a longitudinal decline over time. Second, participants were recruited from a single geriatric ward in a tertiary hospital using convenience sampling, which may limit the generalizability of the findings to other populations, such as community-dwelling older adults, outpatients, or individuals in different healthcare settings. Selection bias may also have been introduced, as only patients who were available and able to complete the assessment were included. Third, pain and some IC domains were measured using self-administered questionnaires, which may lead to reporting bias and underestimation of subclinical impairments. In particular, chronic pain was assessed using a single self-reported question, which may not fully capture the complexity, severity, or multidimensional nature of pain. Future studies using longitudinal designs, more detailed pain assessments, and broader population samples are needed to further validate and extend these findings.

## 5 Conclusions

Chronic pain is significantly associated with IC decline status in older adults with chronic diseases. This relationship remains robust after adjusting for age, health behaviors, and comorbidities. Locomotor and vitality impairments emerged as the most affected domains, highlighting the vulnerability of physical and energy reserves in this population. These findings suggest that routine pain assessment and management should be prioritized to preserve functional capacity and support healthy aging in this population.

## Data Availability

The raw data supporting the conclusions of this article will be made available by the authors, without undue reservation.
